# Anesthesiology Considerations and Management of Venous Air Embolism in Patients in the Semisitting Position: A Single-Center Review

**DOI:** 10.7759/cureus.81093

**Published:** 2025-03-24

**Authors:** Nicholas Drosos, Seth Jacob, Niaman Nazir, Arun S George

**Affiliations:** 1 Anesthesiology, University of Kansas Medical School, Kansas City, USA; 2 Anesthesiology, University of Kansas Medical Center, Kansas City, USA; 3 Population Health, University of Kansas Medical Center, Kansas City, USA

**Keywords:** craniotomy, neurosurgical positioning, semisitting position, transesophageal echocardiography, venous air embolism

## Abstract

Background: The semisitting position is often preferred for neurosurgical procedures requiring access to the cerebellopontine angle or posterior fossa. Despite benefits such as improved venous drainage and reduced intracranial pressure, its use has been controversial due to risks like venous air embolism. Recent advancements in intraoperative monitoring and management have caused renewed interest in this position. This study reviews our institution's experience, focusing on strategies to mitigate complications and improve outcomes in semisitting craniotomies.

Materials and methods: Ninety-four consecutive adult patients who underwent intracranial neurosurgery in the semisitting position were enrolled in the study. The surgery and anesthesiology reports were reviewed to extract data regarding demographics, intraoperative monitoring, and postoperative complications. For each patient who experienced a venous air embolism, an additional set of hemodynamic data was collected. Data management and statistical analyses were performed using Statistical Analysis System (SAS) software (version 9.4, 2023, SAS Institute Inc., Cary, NC, USA).

Results: Venous air embolism occurred in a total of 21 patients (22.34%). Out of these 21 patients, one experienced a venous air embolism that coincided with a decrease in end-tidal carbon dioxide* (*EtCO_2_) of > 3 mmHg. Three patients (3.19%) were transitioned from semisitting to another position, with only one of these due to persistent occult air entrainment despite management efforts.

Conclusion: The semisitting position remains a valuable approach in neurosurgery. We identified multiple factors important in reducing the risk of complications and managing them when they occur. Robust anesthesia guidelines should be developed so that this modality can be used more broadly.

## Introduction

For neurosurgical procedures that require access to the cerebellopontine angle or the posterior fossa, the semisitting position is often preferred [1]. This approach is particularly suitable for managing intracranial tumors such as vestibular schwannomas, which account for eight percent of all intracranial tumors and whose incidence has steadily increased over the past 50 years [2,3]. However, the use of the semisitting position has been a controversial topic for nearly a century [4-6].

On the one hand, the semisitting position has the benefits of improved venous drainage, reduced intracranial pressure, and improved access depending on the location of the tumor. These benefits equate to a better visualization of the surgical field and thereby potentially improve surgical outcomes [7-9]. On the other hand, the semisitting position is associated with a variety of neurologic and cardiopulmonary complications, such as venous air embolism (VAE), tension pneumocephalus, and peripheral nerve injury [10]. Out of these, VAE is the complication associated with the highest risk of morbidity and mortality, so its risk and occurrence must be comprehensively addressed by any surgical team [7].

Due to the high rates of VAE in the semisitting position, surgical teams may elect to place the patient in a prone or lateral position. These alternative positions have been the status quo over the past few decades [7]. However, much has changed in neurosurgery and anesthesiology, such as improvements in intraoperative monitoring and more sensitive methods of identifying VAE. These innovations have encouraged surgical teams to develop new strategies for the semisitting position to improve surgical outcomes, reduce the risk of complications, and diagnose and manage complications early when they occur [10].

Consequently, the semisitting position has garnered renewed interest. Recent literature shows that many surgical teams with experience in this technique report no significant increase in morbidity or mortality when the semisitting position is used [11]. Moreover, studies have reported that operations done in the semisitting position for patients undergoing vestibular schwannoma resection have been associated with better outcomes (e.g., preserved facial nerve function, improved hearing) when compared to similar operations done in the lateral decubitus position [9,12]. In our study, we share our experience with craniotomies in the semisitting position and investigate the frequency and severity of complications we encountered. Our study also focuses on measures we utilized to reduce the risk of these complications and manage them.

## Materials and methods

This retrospective observational study was approved by the Institutional Review Board of the University of Kansas Medical Center on March 6, 2020 (STUDY00145508). Consecutive adult patients who underwent intracranial neurosurgery in the semisitting position at the University of Kansas Medical Center between March 2018 and January 2024 were enrolled in the study. Exclusion criteria were age < 18 and the presence of a large patent foramen ovale (PFO).

The surgery and anesthesiology reports were reviewed retrospectively to extract data regarding demographics, perioperative complications, and intraoperative details, including lines, transesophageal echocardiogram (TEE) probe placement and findings, pinning, patient positioning, medication, hemodynamics (monitored continuously throughout the procedure), and fluid management. For each patient who experienced a VAE, an additional set of hemodynamic data was collected for the 30-minute period immediately following the VAE. The presence of a VAE was defined by visualization of air in the right atrium using TEE.

A risk factor analysis was performed on demographic data to evaluate factors that might contribute to the risk and severity of VAE. Demographic data included patient age, gender, height, weight, American Society of Anesthesiologists (ASA) class, and race/ethnicity (White, Asian, Black, Hispanic, Native Hawaiian or Pacific Islander, and Other (patients who declined to respond to the race/ethnicity on admission) individuals). TEE and intraoperative monitoring data were used to identify VAE, classify severity, and track the efficacy of VAE management by the surgical team. Intraoperative monitoring data included blood pressure, heart rate, end-tidal carbon dioxide, peak pressure, and blood oxygen saturation. Data on postoperative nerve damage was extracted, and for patients who underwent surgery on a vestibular schwannoma, the House-Brackmann score was also recorded.

Preparation for the semisitting position

A majority of patients underwent a preoperative bubble study with a transthoracic echo by a cardiologist to evaluate for intracardiac shunting. For the remaining patients, evaluation was done on the day of surgery by the anesthesia team using a TEE probe after induction of general anesthesia. Patients with a large PFO were excluded. For patients with a medium or small PFO, the surgical team had a discussion regarding the risks and benefits of proceeding in the semisitting position and proceeded accordingly if the benefits outweighed the risks.

In addition to the standard ASA monitors, we inserted a TEE probe to monitor for air bubbles, preferably using the bicaval view in order to visualize the right atrium, superior and inferior vena cava. The four-chamber view was also utilized occasionally. A probe holder was utilized to hold the probe in place. We placed arterial lines in all our patients with the transducer placed at the level of the tragus. We also placed an internal jugular line for aspiration of entrained air in case of a VAE and for central venous pressure (CVP) monitoring. Using the TEE probe, we confirmed that the tip of the catheter was at the junction between the right atrium and the superior vena cava and attached an extension tubing, a three-way stopcock, and a syringe ready to aspirate any air entrained. Total intravenous anesthesia (TIVA) was utilized for all cases using propofol and remifentanil. Neuromonitoring with somatosensory evoked potentials (SSEP) and motor evoked potentials (MEP) was done to monitor patient safety during positioning and throughout the procedure.

Patients were then placed in the semisitting position, which has been described in the literature on many occasions [7,9,10,13]. In summary, the patient was placed in the Trendelenburg position with the head raised to the same level as the feet. The head was supported and secured to prevent undue stress on the neck. Safety straps and padding were applied to support the limbs and prevent pressure injuries.

Intraoperative management

As per our institutional practice, a dedicated anesthesiologist monitored the TEE in the bicaval or four-chamber view for VAE while another member of the anesthesia team attended to the anesthetic management of the patient and charting. Particular attention was given to oxygen saturation (SpO₂), end-tidal carbon dioxide (EtCO₂), tidal volume, heart rate, and blood pressure. Our institution’s internal guidelines recommended maintaining the mean arterial pressure (MAP) between 75-80 mmHg and CVP greater than 5 mmHg.

If air in the heart was visualized via TEE, the anesthesiologist notified the surgical team. The anesthesiologist then compressed the internal jugular vein, and the surgeon attempted to identify the source of air entrainment in the surgical field and apply a sealant such as Surgicel™ (Ethicon US, LLC, New Jersey, USA) to control the venous hemorrhage and air entrainment. If there was a large amount of air in the right atrium, the anesthesiologist attempted to aspirate the air using the syringe connected to the central venous catheter. For instances of VAE that caused hemodynamic changes, a vasopressor (norepinephrine or phenylephrine) was given, and if all these measures were not sufficient to return the patient to hemodynamic stability, the head position would be dropped down, and the surgery would proceed in a prone, supine, or lateral position.

Statistical analysis

Data management and statistical analyses were performed using Statistical Analysis System (SAS) software (version 9.4, 2023, SAS Institute Inc., Cary, NC, USA). Categorical variables were summarized with percentages, and continuous variables were summarized by means and standard deviation. In instances where 50% of the cells had expected counts of less than five, Fisher’s exact test was used to make global comparisons of categorical variables across groups. Two-sided p-values less than 0.05 were considered statistically significant.

## Results

Demographics

A total of 94 patients underwent craniotomy in the semisitting position at our institution during the study. This included 43 males and 51 females. The mean age was 49.5 years. Among the most common race/ethnicity, there were White individuals (81 patients, 86.2%), followed by Asian individuals (five patients, 5.3%), Black individuals (four patients, 4.3%), Hispanic individuals (two patients, 2.1%), Native Hawaiian or Pacific Islander individuals (one patient, 1.1%), and Other individuals (one patient, 1.1%).

The American Society of Anesthesiologists (ASA) scores of patients ranged from two to four, with a score of three being the most common. A PFO was identified in a total of five patients (5.3%) (Table 1). The most common type of pathology seen was vestibular schwannoma (76 patients, 80.9%), followed by pineal parenchymal tumor (seven patients, 7.5%), meningioma (six patients, 6.4%), glioma (two patients, 2.1%), and other (three patients, 3.2%).

**Table 1:** Demographic and perioperative characteristics n: number of patients; ASA: American Society of Anesthesiologists; PFO: patent foramen ovale; VAE: venous air embolism The value in parenthesis is the percentage of the total patients (N = 94) the particular n represents, and for the mean age row, the parenthesis represents the standard deviation.

Characteristics	n (%)
Male (%)	43 (45.7%)
Age in years, mean (SD)	49.5 (14SD)
Race (%)	N/A
White	81 (86.2%)
Asian	5 (5.32%)
Black	4 (4.26%)
Hispanic	2 (2.13%)
Native Hawaiian or Pacific Islander	1 (1.06%)
Other	1 (1.06%)
Tumor type (%)	N/A
Vestibular schwannoma	76 (80.9%)
Pineal parenchymal tumor	7 (7.5%)
Meningioma	6 (6.4%)
Glioma	2 (2.1%)
Other	3 (3.2%)
ASA Class (%)	N/A
1	0 (0%)
2	24 (25.5%)
3	66 (70.2%)
4	4 (4.3%)
5	0 (0%)
6	0 (0%)
PFO (%)	5 (5.3%)
VAE (%)	21 (22.3%)

Analysis of positioning

There were no significant changes when comparing pre-positioning and post-positioning hemodynamic data, including SpO_2_, EtCO_2_, tidal volume, heart rate, and blood pressure. The mean arterial pressure only changed by an average of -3.45 mmHg when patients were positioned. All other metrics changed by a value of less than one unit. Heart rate changed by an average of 0.93, SpO_2_ changed by an average of -0.14, EtCO_2_ changed by an average of -0.17, tidal volume changed by an average of -0.51, and peak pressure changed by an average of 0.41.

A total of three patients (3.2%) were transitioned from semisitting positioning to another position. Two of these patients were converted to the supine position because of a bilateral loss of MEPs during positioning. The other patient was converted to the lateral position due to occult air entrainment that persisted despite packing the surgical area. This patient’s VAE did not lead to a clinically significant reduction in blood pressure or other metrics, but the concern was that the source of air entrainment could not be identified. Air entrainment stopped after the patient was repositioned.

Analysis of venous air embolism

VAE occurred in a total of 21 patients (22.3%), as diagnosed by visualization of air in the right atrium with TEE. Air entrainment was noted only once in these patients, except for four patients who each experienced two separate instances of air entrainment. There was noted to be no significant correlation between the incidence of VAE and patient age, BMI, pre-operative hemoglobin, duration of anesthesia, duration of the operation, or blood loss.

Intraoperative fluid intake was noted to be slightly higher in individuals who experienced VAE. Those who did not experience VAE had an average fluid intake of 952 ml, and those who experienced VAE received an average of 1,324 mL fluid. Another difference was noted in the incidence of VAE by gender. VAE was seen in six out of 37 males (28.6%) and in 15 out of 36 females (71.4%), a notable difference between groups (p = 0.073).

The occurrence of VAE by race/ethnicity was significant. Out of the five Asian patients included in the study, four of them (80%) experienced a VAE. This was significantly higher (p = 0.0123) than the average rate of VAE across all races/ethnicities (22.34%). The single patient in the other race/ethnicity category also experienced a VAE. The next-highest rate of VAE was seen in White, non-Hispanic patients, with 15 out of 81 patients (18.52%) experiencing VAE. In Black patients, one out of four patients (25%) experienced VAE. The two Hispanic patients did not experience VAE, and the single Native Hawaiian or Other Pacific Islander patient did not experience VAE.

In ASA Class 2 patients, four (16.67%) out of 24 experienced VAE. In ASA Class 3 patients, 16 (24.24%) out of 66 experienced VAE. In ASA Class 4 patients, one (25%) out of four patients experienced VAE (Table 2).

**Table 2:** Venous air embolism details The value in parenthesis represents the percentage of total patients who had air entrainment (N = 21). Two-sided p-values < 0.05 were considered statistically significant. n: number of patients; ASA: American Society of Anesthesiologists

Characteristics	n (%)	Statistical test (Value/Probability)	p-value
Sex	N/A	Fisher's exact test (0.04)	0.09
Male	6 (28.6%)	N/A	N/A
Female	15 (71.4%)	N/A	N/A
Race	N/A	Fisher's exact test (0.0003)	0.01
White	15 (71.4%)	N/A	N/A
Asian	4 (19.1%)	N/A	N/A
Black	1 (4.8%)	N/A	N/A
Hispanic	0 (0%)	N/A	N/A
Native Hawaiian or Pacific Islander	0 (0%)	N/A	N/A
Other	1 (4.8%)	N/A	N/A
ASA Class	N/A	Fisher's exact test (0.07)	0.82
1	0 (0%)	N/A	N/A
2	4 (16.67%)	N/A	N/A
3	16 (24.24%)	N/A	N/A
4	1 (25%)	N/A	N/A
5	0 (0%)	N/A	N/A
6	0 (0%)	N/A	N/A

Out of the five patients with a PFO, two (40%) of them experienced VAE. Neither of these two patients experienced a hemodynamically significant VAE.

In the 21 patients who experienced VAE, one patient had to be converted to another position due to persistent occult air entrainment despite packing the entire surgical field. The patient did not experience a decrease in EtCO₂ or any hemodynamic instability.

The Tubingen scale was used to classify VAE based on severity (Table 3) [6]. The scale classifies VAE based on the appearance of air bubbles on TEE, changes in EtCO_2_, and changes in blood pressure. No VAE was observed in 73 patients, who were categorized as Grade 0. Grade I VAE was seen in 14 patients and Grade II VAE was seen in six patients. Only one patient was observed to have a Grade III VAE. None of the patients in our study experienced Grade IV or Grade V air embolisms.

**Table 3:** Tubingen grading scale The value in parenthesis represents the percentage of total patients (N = 94). Two-sided p-values less than 0.05 were considered statistically significant. TEE: transesophageal echocardiography; EtCO_2_: end-tidal carbon dioxide; n: number of patients

Grade	Criteria	n (%)	Statistical test (Value/Probability)	p-value
N/A	N/A	N/A	Fisher's exact test	< .0001
Grade 0	No air bubbles visible on TEE	73 (76.67%)	N/A	N/A
Grade I	Air bubbles visible on TEE	14 (14.89%)	N/A	N/A
Grade II	Air bubbles visible on TEE with a decrease of EtCO_2_ ≤ 3 mmHg	6 (6.38%)	N/A	N/A
Grade III	Air bubbles visible on TEE with a decrease of EtCO_2_ >3 mmHg	1 (1.06%)	N/A	N/A
Grade IV	Air bubbles visible on TEE with decrease of EtCO_2_ >3 mmHg and decrease of mean arterial pressure ≥ 20% or increase of heart rate ≥ 40% (or both)	0 (0%)	N/A	N/A
Grade V	Same as Grade IV, plus causing hemodynamic instability requiring cardiopulmonary resuscitation	0 (0%)	N/A	N/A

Analysis of postoperative nerve complications

New-onset postoperative nerve injury was found in 17 patients (18.09%). In these patients, 15 experienced new-onset injury of the ipsilateral facial nerve, one experienced injury of the trochlear nerve, and one experienced injury of the trigeminal nerve. Nerve injury occurred at a higher rate in patients who experienced VAE (five (23.8%) out of 21 patients) as compared to patients who did not experience VAE (12 (16.4%) out of 73).

The postoperative House-Brackmann scores (HB) were found for 61 patients. A total of 14 patients (22.95%) were HB one, 18 patients (29.50%) were HB two, 13 patients (21.31%) were HB three, six patients (9.84%) were HB four, five patients (8.20%) were HB five, and five patients (8.20%) were HB six.

## Discussion

Our study demonstrates that semisitting craniotomy can be performed safely by experienced surgical and anesthesiology teams. This is consistent with numerous studies that have shown favorable outcomes for surgeries in the semisitting procedure [9,14-16]. We found that while 21 patients (22.34%) had detectable air bubbles on TEE, none experienced hemodynamic instability. Only one patient had a clinically significant VAE, indicated by a greater than 3 mmHg decrease in EtCO₂, which resolved immediately after jugular compression. Our VAE incidence was slightly lower than in other studies using TEE for VAE detection [6,16,17].

**Figure 1:**
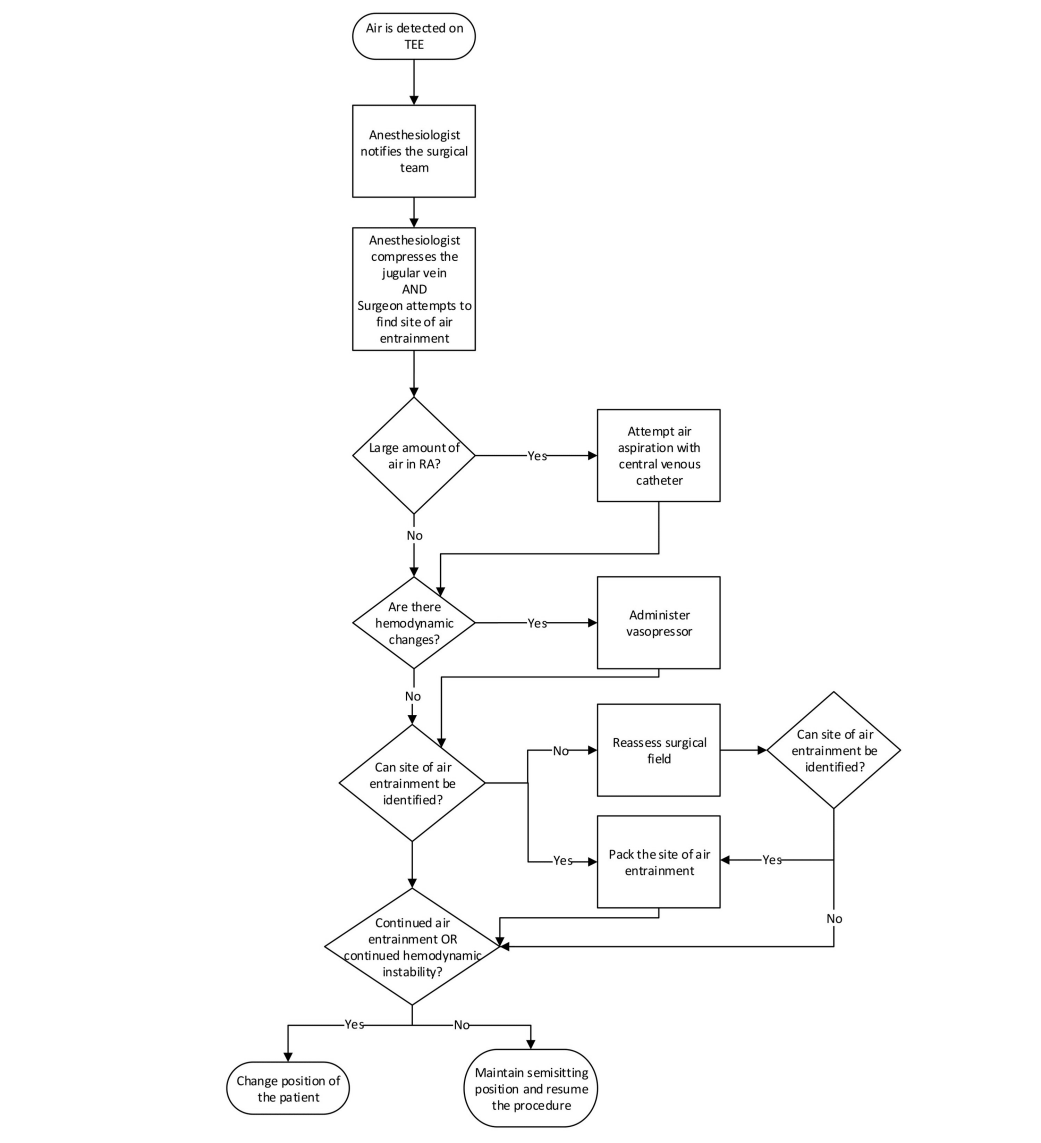
VAE management protocol during sitting craniotomy surgeries VAE: venous air embolism; TEE: transesophageal echocardiogram; RA: right atrium The figure was created by the authors and not reproduced from another source.

We could attribute this to the methodological approach we utilized to ensure the safety and efficacy of the semisitting craniotomy. Each method was carefully selected and implemented to address specific challenges associated with this surgical position. Below, we discuss our protocol and rationale behind each key methodological decision and its impact on our findings (Figure 1).

One significant aspect of our methodology was the use of TEE to monitor for VAE. TEE is widely recognized for its high sensitivity in detecting air bubbles and its real-time visualization of right atrial air entrainment [18]. We believe that TEE is a critical tool for the rapid identification and quantification of even small volumes of air, which is crucial for early diagnosis and management of VAE in the semisitting position. Other benefits of TEE use include ensuring that the central venous catheter is placed in the correct position and testing for the presence of a PFO [10].

To enhance the effectiveness of TEE monitoring, we assigned an additional experienced anesthesiologist to observe the TEE continuously throughout the procedure. This dedicated provider improved the response time in the event of air entrainment, ensuring that corrective measures could be swiftly implemented. The presence of an extra provider focused solely on TEE monitoring underscores the commitment to maximizing patient safety and demonstrates the importance of real-time, specialized oversight for procedures in the semisitting position. However, one unintended consequence of using TEE could be an increased number of VAE false positives, which could lead to unnecessary interruptions during the procedure [15]. TEE use also has several rare but potentially severe side effects, such as bleeding, aspiration, esophageal perforation, and displacement of the glottic tube [19].

Some studies have reported that transthoracic Doppler, although less sensitive than TEE, is adequate for detecting clinically significant VAE [14,15]. Additionally, one group argues that the high cost associated with adding another provider for continuous TEE monitoring is not practical [15]. We acknowledge that these groups reported good outcomes to support these claims. However, we emphasize that the purpose of using TEE is not just to identify clinically significant air entrainment but to discover air entrainment early enough to prevent it from becoming clinically significant. In terms of cost, we contend that early detection and intervention can mitigate the risk of a VAE progressing to a more severe state that would require a more time-consuming and expensive intervention. We believe that this could, in theory, lead to cost savings by preventing the consequences of delayed VAE treatment. This argument could be taken in relation to the cost savings that low molecular weight heparin has in the treatment of inpatients with pulmonary embolism, mainly by reducing the length of hospital stay [20].

Patient positioning is done step by step with the neurosurgery and anesthesia team, ensuring that the patient remains safe hemodynamically as well as anatomically. Immediately after positioning the patient, it was essential to take time to ensure hemodynamic stability.

We also prioritized maintaining access to the patient's neck throughout the procedure to facilitate rapid jugular venous compression if VAE was identified. Jugular venous compression is a maneuver done to increase CVP with the goal of reducing air entrainment [21]. It is also done to help identify the VAE source [22]. By ensuring clear and unobstructed access to the neck, our anesthesia team could quickly implement this intervention, thereby minimizing the potential for further air entrainment. For VAE management, we also placed a central line at the junction between the superior vena cava (SVC) and the right atrium (RA) with the purpose of aspirating entrained air from the RA, which is crucial in preventing hemodynamic instability.

We used TIVA throughout all patients in the study to provide a stable and predictable depth of anesthesia with rapid onset and recovery, which is crucial for maintaining hemodynamic stability. All patients also underwent SSEP and MEP monitoring to ensure the integrity of the nervous system, especially when the neck remained flexed for prolonged periods for the surgery.

As per department practice, only experienced providers were assigned to these cases with the understanding that expertise in recognizing VAE is crucial in this time-sensitive situation. Experienced surgeons and anesthesiologists bring a wealth of knowledge and honed skills that are necessary in detecting subtle signs of air embolism and executing appropriate interventions in a time-effective manner. Their proficiency contributes significantly to the overall safety and success of the procedure, reducing the likelihood of adverse events and improving patient outcomes. However, some drawbacks of this strategy are that it may cause scheduling limitations and that an investment of time in training is required to perform at a high level.

We placed the transducer at the level of the tragus because it better corresponds to cerebral perfusion pressure (CPP), which is crucial for maintaining cerebral oxygenation and preventing ischemic injury, especially in the sitting position where the neck may be in a flexed position for prolonged periods, thus compromising cerebral venous drainage [10].

We also want to address the topic of using the semisitting position for patients with a PFO. The possibility of a paradoxical air embolism in patients with a PFO represents a very high morbidity and mortality risk. Nonetheless, some studies have argued that the semisitting position is safe for patients with a PFO [1,6]. Our study adopted a middle ground by excluding patients with a large-sized PFO while including patients with a small- or medium-sized PFO.

In our study, five patients were noted to have a PFO. Two of those patients experienced VAE, but the air entrainment was not clinically significant in either case. Given the absence of complications in these patients and due to the lack of mortality in patients with PFO in other literature [6], we believe that the semisitting position is reasonably safe for patients with a small- or medium-sized PFO.

Our study also identified a number of possible risk factors for the occurrence of VAE in the semisitting position. We found that the risk of patients with Asian ethnicity to experience a VAE was statistically significant (p = 0.0123). This finding has not been noted in any literature, and no aggressive pathology could be correlated to this observation in our study. However, we want to reiterate that no patients in our study, including these patients, experienced a hemodynamically significant VAE. We also note that in our study, the rate of VAE was higher in women (71.4%) than in men (28.6%) (p = 0.0730). A higher association of VAE in women than in men has not been noted in any other literature. We also analyzed our data set for other possible risk factors, such as lower ASA class, which was reported by one study to be a risk factor [14]. We did not find a lower ASA class or any variables other than Asian ethnicity to be statistically significant risk factors for VAE in our study.

There are multiple limitations to our study. First, we used subjective measures to determine if a patient with a PFO would be placed in the semisitting position. This multifactorial decision varied on a case-by-case basis, so the criteria are not generalizable. Second, we utilized retrospective data, limiting the reliability and completeness of some data. Third, our study included procedures done by multiple different anesthesiologists and neurosurgeons, which could introduce inconsistencies in patient outcomes and the management of intraoperative complications. This variability may affect the comparability of results, especially with TEE skills, and highlight the need for standardized protocols to ensure uniformity in practice. Fourth, some anesthesiologists transitioned from using the TEE probe to using a Doppler ultrasound when the surgeon closed the dura, while others continued monitoring the TEE until TIVA was stopped. This inconsistency may affect the detection and management of late-occurring venous air embolisms, potentially introducing variability in patient outcomes and complicating the interpretation of our results.

## Conclusions

This study demonstrates that the semisitting position for intracranial neurosurgery can be safely utilized. VAEs during sitting craniotomy surgeries have the potential to have catastrophic cardiovascular, pulmonary, and neurological sequelae and have always been a major concern during semisitting craniotomy surgeries. We identified key factors in minimizing the risk and severity of complications for procedures done in this position. These factors include, but are not limited to, the use of TEE monitored by a designated provider, proper positioning and hemodynamic stabilization of the patient, neuromonitoring, the use of TIVA, good communication, and the experience of the entire team. The semisitting craniotomy procedure has several advantages, and a robust anesthesia guideline should be developed so this position can be used more broadly.

## References

[1] Klein J, Juratli TA, Weise M, Schackert G (2018). A systematic review of the semi-sitting position in neurosurgical patients with patent foramen ovale: how frequent is paradoxical embolism?. World Neurosurg.

[2] Carlson ML, Link MJ (2021). Vestibular schwannomas. N Engl J Med.

[3] Marinelli JP, Lohse CM, Carlson ML (2018). Incidence of vestibular schwannoma over the past half-century: a population-based study of Olmsted County, Minnesota. Otolaryngol Head Neck Surg.

[4] De Martel T (1930). The sitting position in neurosurgery: a critical appraisal. Surg Gynecol Obstet.

[5] Safdarian M, Safdarian M, Chou R, Hashemi SM, Rahimi-Movaghar V (2017). A systematic review about the position-related complications of acoustic neuroma surgery via suboccipital retrosigmoid approach: sitting versus lateral. Asian J Neurosurg.

[6] Feigl GC, Decker K, Wurms M, Krischek B, Ritz R, Unertl K, Tatagiba M (2014). Neurosurgical procedures in the semisitting position: evaluation of the risk of paradoxical venous air embolism in patients with a patent foramen ovale. World Neurosurg.

[7] Porter JM, Pidgeon C, Cunningham AJ (1999). The sitting position in neurosurgery: a critical appraisal. Br J Anaesth.

[8] Gracia I, Fabregas N (2014). Craniotomy in sitting position: anesthesiology management. Curr Opin Anaesthesiol.

[9] Schackert G, Ralle S, Martin KD (2021). Vestibular schwannoma surgery: outcome and complications in lateral decubitus position versus semi-sitting position-a personal learning curve in a series of 544 cases over 3 decades. World Neurosurg.

[10] Tufegdzic B, Lamperti M, Siyam A, Roser F (2021). Air-embolism in the semi-sitting position for craniotomy: a narrative review with emphasis on a single centers experience. Clin Neurol Neurosurg.

[11] Vychopen M, Arlt F, Güresir E, Wach J (2023). How to position the patient? A meta-analysis of positioning in vestibular schwannoma surgery via the retrosigmoid approach. Front Oncol.

[12] Roessler K, Krawagna M, Bischoff B (2016). Improved postoperative facial nerve and hearing function in retrosigmoid vestibular schwannoma surgery significantly associated with semisitting position. World Neurosurg.

[13] Jadik S, Wissing H, Friedrich K, Beck J, Seifert V, Raabe A (2009). A standardized protocol for the prevention of clinically relevant venous air embolism during neurosurgical interventions in the semisitting position. Neurosurgery.

[14] Al-Afif S, Elkayekh H, Omer M (2022). Analysis of risk factors for venous air embolism in the semisitting position and its impact on outcome in a consecutive series of 740 patients. J Neurosurg.

[15] Ammirati M, Lamki TT, Shaw AB, Forde B, Nakano I, Mani M (2013). A streamlined protocol for the use of the semi-sitting position in neurosurgery: a report on 48 consecutive procedures. J Clin Neurosci.

[16] Scheller C, Rampp S, Tatagiba M (2020). A critical comparison between the semisitting and the supine positioning in vestibular schwannoma surgery: subgroup analysis of a randomized, multicenter trial. J Neurosurg.

[17] Ganslandt O, Merkel A, Schmitt H, Tzabazis A, Buchfelder M, Eyupoglu I, Muenster T (2013). The sitting position in neurosurgery: indications, complications and results. a single institution experience of 600 cases. Acta Neurochir (Wien).

[18] Mirski MA, Lele AV, Fitzsimmons L, Toung T (20071). Diagnosis and treatment of vascular air embolism. Anesthesiology.

[19] Daniel WG, Erbel R, Kasper W (1991). Safety of transesophageal echocardiography. A multicenter survey of 10,419 examinations. Circulation.

[20] Aujesky D, Smith KJ, Cornuz J, Roberts MS (2005). Cost-effectiveness of low-molecular-weight heparin for treatment of pulmonary embolism. Chest.

[21] Eckle VS, Neumann B, Greiner TO, Wendel HP, Grasshoff C (2015). Intrajugular balloon catheter reduces air embolism in vitro and in vivo. Br J Anaesth.

[22] Losasso TJ, Muzzi DA, Cucchiara RF (1992). Jugular venous compression helps to identify the source of venous air embolism during craniectomy in patients in the sitting position. Anesthesiology.

